# Optimal Trajectory Planning of the Variable-Stiffness Flexible Manipulator Based on CADE Algorithm for Vibration Reduction Control

**DOI:** 10.3389/fbioe.2021.766495

**Published:** 2021-10-08

**Authors:** Qiang Cheng, Wenxiang Xu, Zhifeng Liu, Xiaolong Hao, Yi Wang

**Affiliations:** ^1^ Institute of Advanced Manufacturing and Intelligent Technology, Beijing University of Technology, Beijing, China; ^2^ Beijing Key Laboratory of Advanced Manufacturing Technology, Beijing University of Technology, Beijing, China; ^3^ Department of Stomatology, General Hospital of the PLA, Beijing, China

**Keywords:** variable-stiffness flexible manipulator, control design, vibration reduction, trajectory planning, CADE optimization algorithm

## Abstract

Robotic manipulators are widely used for precise operation in the medical field. Vibration suppression control of robotic manipulators has become a key issue affecting work stability and safety. In this paper an optimal trajectory planning control method to suppress the vibration of a variable-stiffness flexible manipulator considering the rigid-flexible coupling is proposed. Through analyzing the elastic deformation of the variable-stiffness flexible manipulator, a distributed dynamic physical model of the flexible manipulator is constructed based on the Hamilton theory. Based on the mathematical model of the system, the design of the vibration damping controller of the flexible manipulator is proposed, and the control system with nonlinear input is considered for numerical analysis. According to the boundary conditions, the vibration suppression effect of the conventional and the variable-stiffness flexible manipulator is compared. The motion trajectory of the variable-stiffness flexible manipulator and compare the vibration response from different trajectories. Then, with minimum vibration displacement, minimum energy consumption and minimum trajectory tracking deviation as performance goals, the trajectory planning of the variable-stiffness flexible manipulator movement is carried out based on the cloud adaptive differential evolution (CADE) optimization algorithm. The validity of the proposed trajectory planning method is verified by numerical simulation.

## Introduction

Robotic manipulator plays an important role in medical diagnosis, due to the advantages of fast running speed, high accuracy, and low energy consumption. The dynamic analysis and vibration suppression control of the flexible robot manipulator system had attracted the attention to many scholars (Pratiher and Dwivedy, 2007; H. Moharam et al., 2013; Qiu et al., 2015; Cao and Liu, 2019; He et al., 2019; Jiang et al., 2021). The flexible manipulator is a dynamic system with strong nonlinearity and strong rigid-flexible coupling. The robot manipulators are generally considered to be conventional Eular-Bemolulli beams and Timoshcnko beams of equal cross-section (Timoshcnko, 1922; Benosman and Le Vey, 2004; Dwivedy and Eberhard, 2006). In the existing research on vibration control of flexible manipulators, Euler-Bernoulli beam theory is mostly used for theoretical modeling (Mladenova and Rashkov, 2004).

In recent years, scholars’ main research has focused on homogeneous and continuum manipulator, rectangular thin manipulator, functionally graded manipulator, and rotating flexible manipulator with additional mass (Cai et al., 2005; Cai and Lim, 2008; Dupont et al., 2010; Fan, 2012; Li et al., 2014; Chen et al., 2018). Zaher and Megahed (2015) described the deformation of the flexible manipulator using the hypothetical modal method and established a relatively complete dynamic model of the flexible manipulator. Macnab et al. (2004) studied the effect of the centralized mass method in the description of deformation, and verified through experiments that the centralized mass method has better processed results of the complex shape of the manipulator, but the positioning accuracy is low. In terms of modeling theory, Moallem et al. (2015) established a dynamic model of a flexible manipulator with end mass using Hamilton’s principle, and verified the effectiveness of Hamilton’s principle through numerical simulation. Herrnstadt and Menon (2016) developed a single degree of freedom elbow orthosis and performed a linear modeling on the suppression system. The linear model has been widely used to design the control technology of homogeneous flexible manipulators (Huang and Ji, 2020). However, less work has been done on the nonlinear modeling of variable stiffness manipulators.

At present, there are many researches on physical components and control optimization methods for suppressing vibration of flexible manipulator (Diken, 2000; Jinqiao et al., 2010; Guo et al., 2016; Wilbanks and Leamy, 2019; Chen et al., 2021; Niu et al., 2021; Zhang et al., 2021). Korayem and Ghariblu (2004), Ghariblu and Korayem (2006), Korayem et al. (2011), and Korayem et al. (2013) proposed an open-loop optimal control method to generate the optimal trajectory of a flexible mobile manipulator in point-to-point motion, so that the robot can bear the maximum load between two designated terminal positions. For the single-link flexible arm mounted on the base (Abe, 2009; Abe and Komuro, 2012; Abe, 2013), proposed a point-to-point trajectory planning algorithm. The cycloid function is used as the benchmark of motion trajectory interpolation, and the end residual amplitude is minimized as the goal for optimization, and a good vibration suppression effect is achieved. Boscariol and Gasparetto (2013) used the finite element method to establish a dynamic model of the planar flexible manipulator, and then used the indirect method to plan the trajectory of the planar flexible manipulator. Korayem et al. (2009) established an optimization model of boundary value constraints based on Pontryagin theory, and obtained the vibration suppression trajectory of the point-to-point control of the flexible manipulator. Fairs et al. (2009) used fourth-order polynomial motion trajectory and soft motion trajectory respectively, and took the loss energy in the motion process as the fitness function, and used genetic algorithm to optimize the motion trajectory of the two-link flexible manipulator to suppress residual vibration. Heidari et al. (2013) and others established a nonlinear finite element dynamic model of a three-dimensional flexible manipulator, and based on Pontryagin theory to use optimal control to obtain the optimal trajectory with minimum energy and minimum vibration. Boscariol and Gasparetto (2013) proposes a point-to-point trajectory plan method of minimum actuator jerks and vibrations. However, they did not consider the effects of variable stiffness, amplitude, energy consumption and trajectory approximation errors at the same time. Moreover, there are few researches on vibration suppression of flexible manipulators with variable stiffness rigid-flexible coupling, which brings challenges to the design of control methods.

In this paper, the problem of vibration suppression control of a variable-stiffness flexible manipulator in the presence of nonlinear input is studied. There are three contributions of this paper.

1) The PDE model of the variable-stiffness flexible manipulator is given in the presence of nonlinear input, which has better dynamic characteristics than the conventional manipulator.
2) A feedback controller of a manipulators that can realize joint angled control and suppress boundary vibration is proposed, in which the manipulator adopts a variable stiffness design.
3) The global optimization performance of the traditional optimization algorithm is improved by the CADE optimization algorithm, and then the minimum vibration displacement, the minimum energy consumption and the minimum trajectory tracking deviation are the performance goals, and the trajectory planning motion control of the variable stiffness flexible manipulator is carried out.

The remainder of this article is structured as follows. The PDE dynamic model for the variable-stiffness flexible manipulator is presented in *Dynamic Modeling of Variable-Stiffness Flexible Manipulator* section. A control method of vibration suppression of the variable-stiffness flexible manipulator is proposed to the nonlinear input in *Control Design of the Flexible Manipulator* section. Numerical Analysis of the flexible manipulator is carried out in *Numerical Analysis of the Flexible Manipulator* section. Numerical simulation results of optimization of vibration suppression trajectory are shown in *Optimization of Vibration Suppression Trajectory* section and conclusions are given in *Conclusion* section. The results show that the trajectory planning effect of the variable-stiffness robotic manipulator based on the differential evolution algorithm is better. The robotic manipulator system moves under the optimal vibration suppression trajectory and has smaller residual vibration.

## Dynamic Modeling of Variable-Stiffness Flexible Manipulator

**FIGURE 1:**
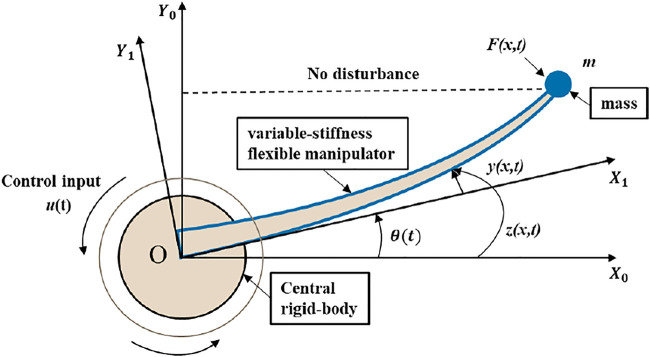
Variable-stiffness flexible manipulator system.

For the variable-stiffness flexible manipulator system driven by the central rigid body, the analytical model is considered shown in Figure 1. The flexible mechanical manipulator is fixed on a central rigid body rotating around a fixed axis in a cantilever manner. The end load is considered as a mass *m*, and the influence of the mass size of the system is ignored. When working, the flexible manipulator is driven by the central rigid body to rotate around the vertical axis O in the horizontal plane.

### Preliminaries

In order to facilitate the subsequent analysis, we propose the following lemmas, explanations and hypotheses for the formula derivation of the research.

Remark 1 For clarify, the notation (∗)′=∂(∗)/∂t , (∗)″=∂(∗)/∂t , ${\left(*\right)}_{x}=\partial \left(*\right)/\partial x$ , ${\left(*\right)}_{xx}=\partial^{2}\left(*\right)/\partial x^{2}$ , ${\left(*\right)}_{xxxx}=\partial^{4}\left(*\right)/\partial x^{4}$ , ${\left(*\right)}_{x}^{\prime }=\partial^{2}\left(*\right)/\partial t\partial x$ , ${\left(*\right)}_{xx}^{\prime }=\partial^{3}\left(*\right)/\partial t\partial x^{2}$

Lemma 1 The length of the flexible manipulator is L, let $\varphi_{1}\left(x,t\right)$ , $\varphi_{2}\left(x,t\right)\in R$ with x∈[0,L] and t∈[0,∞] . Then the following inequalities hold as (Rahn, 2002)

$$\left|\varphi_{1}\left(x,t\right)\varphi_{2}\left(x,t\right)\right|\leq \frac{1}{\delta }\varphi_{1}^{2}\left(x,t\right)+\delta \varphi_{2}^{2}\left(x,t\right)$$ (1)

Lemma 2 Let ϕ(x,t)∈R be a a function defined on x∈[0,L] and t∈[0,∞] . Then the following inequalities hold as (Rahn, 2002)

$$\left\{ \begin{array}{l} \int_{0}^{L}\phi^{2}\left(x,t\right)\leq L^{2}\int_{0}^{L}\phi_{x}^{2}\left(x,t\right)dx\\ \phi^{2}\left(x,t\right)\leq L\int_{0}^{L}\phi_{x}^{2}\left(x,t\right)dx \end{array}\right.$$ (2)

Assumption 1 In this paper, the effect of gravity is ignored in the established physical model. Since both the rotational movement and elastic vibration of the flexible manipulator occurs to the horizontal plane, and the length of the mechanical manipulator is much larger than its cross-sectional width and height, it is assumed to be an Eider-Bernoulli beam.

### Dynamics Analysis of the Flexible Manipulator

As shown in Figure 1, $X_{0}OY_{0}$ is defined as the inertial coordinate of the system, the coordinate system $X_{1}OY_{1}$ is a follow-up coordinate system fixed on the flexible manipulator, and the *X*_*1*_ axis is always tangent to the root of the flexible manipulator. The offset of the flexible manipulator during the movement is z(x,t) , and the displacement of the flexible manipulator is z(x,t)=y(x,t)+x⋅θ(t) , where y(x, t) and θ(t) respectively indicate the elastic deflection of the flexible manipulator and the angular position of the flexible manipulator.

The kinetic energy $E_{k}$ of the manipulator includes the rotational kinetic energy $E_{k1}$ of the central rigid body, the kinetic energy $E_{k2}$ of the flexible manipulator and the kinetic energy $E_{k3}$ of the mass of the flexible manipulator. The relationship between them can be given as $E_{k}=E_{k1}+E_{k2}+E_{k3}$ , $E_{k1}$ , $E_{k2}$ , $E_{k3}$ can be written as

$$\left\{ \begin{array}{l} E_{k1}\left(t\right)=\frac{1}{2}I_{h}\theta \prime {\left(t\right)}^{2}\\ E_{k2}\left(t\right)=\frac{1}{2}\int_{0}^{L}\rho_{L}A\left(x\right)z\prime^{2}\left(x,t\right)dx\\ E_{k3}\left(t\right)=\frac{1}{2}mz\prime^{2}\left(L,t\right) \end{array}\right.$$ (3)

Where $I_{h}$ represents the moment of inertia of the central rigid body, $\rho_{L}$ is the density of the flexible manipulator, A(x) is the cross-sectional area of the flexible manipulator, which also changes with the length *x*. θ(t) represents the actual rotation angle of the flexible manipulator, and z(x,t) is the absolute displacement of t the flexible manipulator in the $X_{0}OY_{0}$ coordinate system.

The potential energy *Ep* of the flexible arm system is expressed as follows

$$E_{p}\left(t\right)=\frac{1}{2}\int_{0}^{L}E_{L}I\left(x\right)y_{xx}^{2}\left(x,t\right)dx+\frac{1}{2}T_{L}\int_{0}^{L}{\left[y_{x}\left(x,t\right)\right]}^{2}dx$$ (4)

Where $E_{L}$ is the elastic modulus of the flexible manipulator, $T_{L}$ is tension of the flexible manipulator, and I(x) is the moment of inertia that changes with the length *x*. y(x,t) is the elastic vibration displacement of the flexible arm in the $X_{1}OY_{1}$ coordinate system.

The control moment *u(x,t)* is added to drive the flexible manipulator system, *F(L,t)* is the input torque of the mass of the flexible manipulator, and its non-conservative force work $W_{c}\left(t\right)$ can be expressed as

$$W_{c}\left(t\right)=u\left(x,t\right)\cdot \theta \left(t\right)+F\left(L,t\right)z\left(L,t\right)$$ (5)

The continuous mass distribution and continuous stiffness distribution characteristics of the flexible manipulator are considered. According to Hamilton’s principle Eq. 37, the variational equation of the flexible manipulator is defined as

$$\int_{t_{1}}^{t_{2}}\left(\delta E_{k}\left(t\right)-\delta E_{p}\left(t\right)+\delta W_{c}\left(t\right)\right)dt=0,\forall \left(x,t\right)\in \left(0,L\right)\times \left[0,t_{\max}\right]$$ (6)

Combining Eqs 3, 6, the variational formula for the kinetic energy $E_{k}\left(t\right)$ of the flexible manipulator is simplified based on the rule of integration as

$$\int_{t_{1}}^{t_{2}}\delta E_{k}\left(t\right)dt=-\int_{t_{1}}^{t_{2}}I_{h}\theta \prime \prime \left(t\right)\delta \theta \left(t\right)dt-\int_{t_{1}}^{t_{2}}\int_{0}^{L}\rho_{L}A\left(x\right)z\prime \prime \left(x,t\right)\delta z\left(x,t\right)dxdt-\int_{t_{1}}^{t_{2}}mz\prime \prime \left(L,t\right)\delta z\left(L,t\right)dt$$ (7)

Combining Eqs 4, 6, the variational formula for the potential energy $E_{p}\left(t\right)$ of the flexible manipulator is simplified based on the rule of integration as

$$\begin{array}{l} \int_{t_{1}}^{t_{2}}\delta E_{p}\left(t\right)dt=\int_{t_{1}}^{t_{2}}\left[T_{L}y_{x}\left(L,t\right)-E_{L}I\left(x\right)y_{xxx}\left(L,t\right)\right]\delta y\left(L,t\right)dt\\ +\int_{t_{1}}^{t_{2}}\left[E_{L}I\left(x\right)y_{xxx}\left(0,t\right)-T_{L}y_{x}\left(0,t\right)\right]\delta y\left(0,t\right)dt\\ +E_{L}I\left(x\right)\int_{t_{1}}^{t_{2}}y_{xx}\left(L,t\right)\delta y_{x}\left(L,t\right)dt\\ +\int_{t_{1}}^{t_{2}}\int_{0}^{L}\left[E_{L}I\left(x\right)y_{xxxx}\left(x,t\right)-T_{L}y_{xx}\left(x,t\right)\right]\delta y\left(x,t\right)dxdt \end{array}$$ (8)

Combining Eqs 5, 6, the variational formula for the non-conservative force work $W_{c}\left(t\right)$ of the flexible manipulator is simplified based on the rule of integration as

$$\int_{t_{1}}^{t_{2}}\delta W_{c}dt=\delta \int_{t_{1}}^{t_{2}}\left(u\left(x,t\right)\theta \left(t\right)dt+F\left(x,t\right)z\left(L,t\right)\right)dt$$ (9)

Based on Eqs 6, 7, the boundary conditions of the flexible manipulator are processed. The central rigid body is the fixed end, and its elastic displacement and elastic angular displacement are both zero. The end of the flexible manipulator is in a free state, and its bending moment and shear force are both zero. Then the boundary conditions of the flexible manipulator can be obtained as

$$\left\{ \begin{array}{l} y\left(0,t\right)=0\\ y_{x}\left(0,t\right)=0\\ z{\prime \prime }_{x}\left(0,t\right)=\theta \prime \prime \left(t\right)\\ E_{L}I\left(L\right)y_{xx}\left(L,t\right)=0\\ mz\prime \prime \left(L,t\right)+T_{L}y_{x}\left(L,t\right)-E_{L}I\left(L\right)y_{xxx}\left(L,t\right)-F\left(x,t\right)=0 \end{array}\right.$$ (10)

Property 1: If $\forall \left(x,t\right)\in \left(0,L\right)\times [0,t_{\max})$ , the kinetic energy of the system described by Eq. 3 is bounded, then the state z′(x,t) , $z\prime_{x}\left(x,t\right)$ , $z\prime_{xx}\left(x,t\right)$ , $z\prime_{xxx}\left(x,t\right)$ related to it is also bounded in the corresponding range.

Property 2: If $\forall \left(x,t\right)\in \left(0,L\right)\times [0,t_{\max})$ , the kinetic energy of the system described by Eq. 3 is bounded, then the state $y_{xx}\left(x,t\right)$ , $y_{xxx}\left(x,t\right)$ , $y_{xxxx}\left(x,t\right)$ related to it is also bounded in the corresponding range.

## Control Design of the Flexible Manipulator

### Design of Control Method

According to the analytical model without external disturbance, the following variable-stiffness flexible manipulator system with governing equation as

$$E_{L}I\left(x\right)\cdot z_{xxxx}\left(x,t\right)+\rho_{L}A\left(x\right)\cdot y\prime \prime \left(x,t\right)=T_{L}\cdot y_{xx}\left(x,t\right)$$ (11)

Where $E_{L}$ is the elastic modulus of the flexible manipulator, $T_{L}$ is tension of the flexible manipulator, and I(x) is the moment of inertia that changes with the length *x*.

The control objective is to build a distributed control *u(t)* to ensure that system state *y(x, t)* can track the variable reference trajectory $\theta_{d}\left(t\right)$ without violation of desired constraint. The force balance relationship of the boundary can be expressed as

$$u\left(t\right)=I_{h}\theta \prime \prime \left(t\right)-E_{L}Iy_{xx}\left(0,t\right)-T_{L}y\left(L,t\right)$$ (12)

A nonlinear boundary input is applied to the end of the manipulator, and control is performed at the end of the manipulator to adjust the vibration of the manipulator, so that the system tends to stabilize faster. According to the boundary Eq. 10, the nonlinear boundary input *F(x,t)* can be obtained as

$$F\left(x,t\right)=mz\prime \prime \left(L,t\right)+T_{L}y_{x}\left(L,t\right)-E_{L}I\left(L\right)y_{xxx}\left(L,t\right)$$ (13)

When the kinetic energy, the potential energy of the flexible manipulator, and the kinetic energy of the mass are the smallest, the elastic deformation y(x, t) of the flexible manipulator is the smallest. Through considering tracking error and tracking error rate of change, the Lyapunov function is constructed as

$$V\left(t\right)=V_{1}\left(t\right)+V_{2}\left(t\right)+V_{3}\left(t\right)$$ (14)

Where $V_{1}\left(t\right)$ is the sum of the kinetic energy and potential energy of the flexible manipulator, and represents an index for restraining the bending deformation and bending change rate of the flexible manipulator. $V_{2}\left(t\right)$ represents the control error index and the kinetic energy of the mass. $V_{3}\left(t\right)$ is the cross auxiliary term. Then $V_{1}\left(t\right)$ , $V_{2}\left(t\right)$ and $V_{3}\left(t\right)$ are defined as

$$\left\{ \begin{array}{l} V_{1}\left(t\right)=\left(\int_{0}^{L}\rho_{L}A\left(x\right)z{'}^{2}\left(x,t\right)dx+E_{L}I\left(x\right)\int_{0}^{L}y_{xx}^{2}\left(x,t\right)dx+T_{L}\int_{0}^{L}y_{x}^{2}\left(x,t\right)dx\right)/2\\ V_{2}\left(t\right)=\left(I_{h}e'{\left(t\right)}^{2}+k_{1}e{\left(t\right)}^{2}+mz{'}^{2}\left(L,t\right)\right)/2\\ V_{3}\left(t\right)=\alpha A\left(x\right)\int_{0}^{L}y\left(x,t\right)z'\left(x,t\right)dx+\beta A\left(x\right)\int_{0}^{L}\left(L-x\right)y_{x}\left(x,t\right)z'\left(x,t\right)dx \end{array}\right.$$ (15)

**FIGURE 2:**
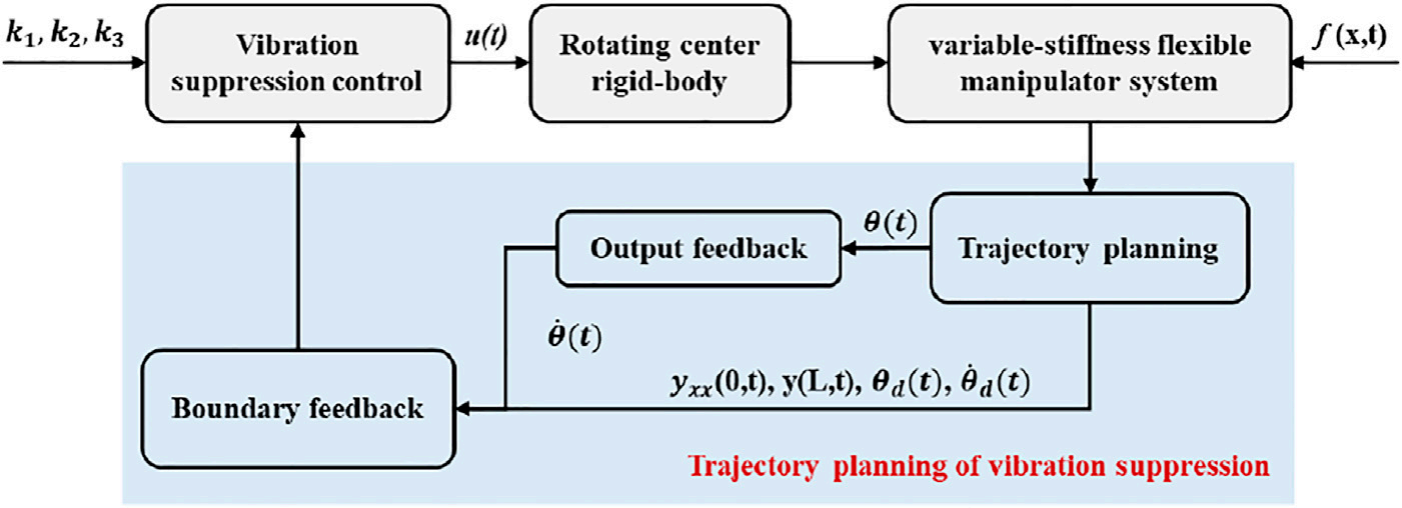
Block diagram of control with trajectory planning for the flexible manipulator.

Where *k*_1_ is the gain related to the controller, and $k_{1}>0$ , α>0 , β>0 . Then the vibration suppression control strategy design is shown in Figure 2.

In the absence of external disturbances, the controller *u(t)* is designed to suppress vibration and track the trajectory $\theta_{d}\left(t\right)$ . Combining the control equations, boundary conditions and candidate Lyapunov function, through calculation and deduction, the controller of the variable-stiffness flexible manipulator system is designed as

$$u\left(t\right)=-k_{1}\left(\theta \left(t\right)-\theta_{d}\left(t\right)\right)-k_{2}\left(\theta '\left(t\right)-\theta {'}_{d}\left(t\right)\right)-k_{3}y\left(L,t\right)$$ (16)

Where *k*_2_ is the gain related to the controller, and $k_{2}>0$ , $k_{3}>0$ .

### Analysis of System Stability and Boundedness

Lemma 3 The boundedness of the Lyapunov function Eq. 15 is given as

$$0\leq \lambda_{1}\left(V_{1}\left(t\right)+V_{2}\left(t\right)\right)\leq V\left(t\right)\leq \lambda_{2}\left(V_{1}\left(t\right)+V_{2}\left(t\right)\right)$$ (17)

where $\lambda_{1},\lambda_{2}>0$ .

Proof For the cross-phase Eq. 15, the following inequality can be obtained as

$$\begin{array}{l} \left|V_{3}\left(t\right)\right|\leq \alpha A\left(x\right)\left(\int_{0}^{L}y^{2}\left(x,t\right)dx+\int_{0}^{L}z{'}^{2}\left(x,t\right)dx\right)+\beta A\left(x\right)L\left(\int_{0}^{L}y_{x}^{2}\left(x,t\right)dx+\int_{0}^{L}z{'}^{2}\left(x,t\right)dx\right)\\ \;\leq \left(\alpha A\left(x\right)L^{2}+\beta A\left(x\right)L\right)\int_{0}^{L}y_{x}^{2}\left(x,t\right)dx+\left(\alpha A\left(x\right)+\beta A\left(x\right)L\right)\int_{0}^{L}z{'}^{2}\left(x,t\right)dx\\ \;\leq \xi_{1}V_{1}\left(t\right) \end{array}$$ (18)

Where $\xi_{1}=\max\left\{\frac{\alpha L^{2}+\beta L}{T_{L}},\frac{\alpha +\beta L}{\rho }\right\}$ .

Then $V_{3}\left(t\right)$ can be obtained as

$$0\leq \left(1-\xi_{1}\right)V_{1}\left(t\right)+V_{2}\left(t\right)\leq V\left(t\right)\leq \left(1+\xi_{1}\right)V_{1}\left(t\right)+V_{2}\left(t\right)$$ (19)

Therefore, V(t) can be obtained as

$$0\leq \lambda_{1}\left(V_{1}\left(t\right)+V_{2}\left(t\right)\right)\leq V\left(t\right)\leq \lambda_{2}\left(V_{1}\left(t\right)+V_{2}\left(t\right)\right)$$ (20)

Where $\lambda_{1}=\min\left(1-\xi_{1},1\right)$ and $\lambda_{2}=\max\left(1+\xi_{2},1\right)$ are two positive constants.

Lemma 4 The time derivative of the Lyapunov function Eq. 14 is proved to be bounded as

V(t)≤−λV(t) (21)

Proof Differentiating Eq. 14 with respect to time, V′(t) is obtained as

$$V\prime \left(t\right)=V\prime_{1}\left(t\right)+V\prime_{2}\left(t\right)+V\prime_{3}\left(t\right)$$ (22)

The error information e(t) , e′(t) , e″(t) of the angle can be obtained as

$$\left\{ \begin{array}{l} e\left(t\right)=\theta \left(t\right)-\theta_{d}\left(t\right)\\ e\prime \left(t\right)=\theta \prime \left(t\right)-\theta \prime_{d}\left(t\right)\\ e\prime \prime \left(t\right)=\theta \prime \prime \left(t\right)-\theta {\prime \prime }_{d}\left(t\right) \end{array}\right.$$ (23)

Substituting boundary condition Eqs 10–15 into Eq. 22, $V\prime_{1}\left(t\right)$ can be obtained as

$$V\prime_{1}\left(t\right)=-E_{L}I\prime \left(x\right)z\prime \left(L,t\right)y_{xxx}\left(L,t\right)-E_{L}I\prime \left(x\right)y_{xx}\left(0,t\right)\theta \prime \left(t\right)+T_{L}\int_{0}^{L}\theta \prime \left(t\right)xy_{xx}\left(x,t\right)dx$$ (24)

Then $V\prime_{2}\left(t\right)$ can be obtained as

$$V\prime_{2}\left(t\right)=e\prime \left(t\right)\left[I_{h}e\prime \prime \left(t\right)+k_{1}e\left(t\right)\right]+z\prime \left(L,t\right)mz\prime \prime \left(L,t\right)$$ (25)

Then $V\prime_{3}\left(t\right)$ can be obtained as

$$V{'}_{3}\left(t\right)=\alpha A\prime \left(x\right)\int_{0}^{L}\left[y\prime \left(x,t\right)z\prime \left(x,t\right)+y\left(x,t\right)z\prime \prime \left(x,t\right)\right]dx+\beta A\prime \left(x\right)\int_{0}^{L}\left(L-x\right)\left[y\prime_{x}\left(x,t\right)z\prime \left(x,t\right)+y_{x}\left(x,t\right)z\prime \prime \left(x,t\right)\right]dx$$ (26)

Combining Eqs 12, 16, 22, 24–26, based on Lemma 1 and Lemma 2, V′(t) can be obtained as

$$V\prime \left(t\right)\leq -\gamma_{1}\int_{0}^{L}z{'}^{2}\left(x,t\right)dx-\gamma_{2}\int_{0}^{L}y_{xx}^{2}\left(x,t\right)dx-k_{1}e^{2}\left(t\right)-\gamma_{3}e{'}^{2}\left(t\right)-\gamma_{4}y_{xx}^{2}\left(0,t\right)-\gamma_{5}\int_{0}^{L}y_{x}^{2}\left(x,t\right)dx$$ (27)

Where

$$\left\{ \begin{array}{l} \gamma_{1}=-\beta /2-\beta L\sigma_{5}-L\beta \sigma_{6}-\alpha -\alpha L/\sigma_{7}\\ \gamma_{2}=EI\left(x\right)\alpha /\rho -T_{L}L^{3}/\sigma_{1}-\left(T-k_{3}\right)L^{3}/\sigma_{4}-3\beta E_{L}I\left(x\right)/2\rho \\ \gamma_{3}=k_{2}-T\sigma_{1}-E_{L}I\left(x\right)/\sigma_{2}-\beta L/\sigma_{5}-\alpha L^{2}\sigma_{7}\\ \gamma_{4}=\beta E_{L}I\left(x\right)L/2\rho -E_{L}I\left(x\right)/\sigma_{3}-\alpha E_{L}I\left(x\right)\sigma_{2}\\ \gamma_{5}=\alpha /\rho -\beta T/2\rho \end{array}\right.$$ (28)

The parameters are chosen appropriately to make sure that $\sigma_{n}>0$ , n = 1–6.

Then combining Lemma 3 and Eq. 27, V′(t) can be obtained as

$$V\prime \left(t\right)\leq -\lambda_{3}\left[V_{1}\left(t\right)+V_{2}\left(t\right)\right]\leq -\lambda V\left(t\right)$$ (29)

where $\lambda_{3}=\min\left\{\frac{2\gamma_{1}}{\rho },\frac{2\gamma_{2}}{E_{L}I\left(L\right)},\frac{2k_{1}}{k_{1}+k_{2}}\right\}$ , and $\lambda =\lambda_{3}/\lambda_{2}>0$ .

With Lyapunov direct method and based on Lemmas 1 and 2, the stability of the system with the proposed control law is analyzed. According to the analysis result, it can be found that the control system is a closed loop system and the system is stable. When the appropriate control gain parameters are selected, the system vibration state and angle tracking error will eventually converge. So as to achieve the purpose of restraining the elastic vibration during the movement towards the system and driving the arm of a predetermined angle.

## Numerical Analysis of the Flexible Manipulator

**FIGURE 3:**
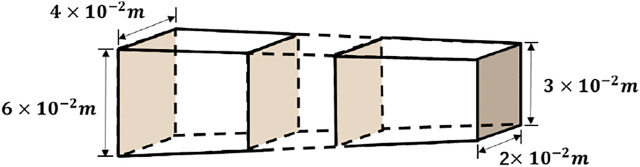
Numerical simulation structural drawing.

In order to investigate the reliability of the control model, a specimen with variable stiffness was designed for dynamic testing. The dynamic tests were carried out on specimens of the shape shown in Figure 3.

The material of the variable-stiffness flexible manipulator is aluminum alloy, and the central rigid body is driven by a private motor. The material properties of the variable-stiffness flexible manipulator system are given in Table 1. It is assumed that the joints of the flexible manipulator system completely track the trajectory during the movement, the whole movement process of the flexible manipulator is numerically simulated.

**TABLE 1:** Parameters of the variable-stiffness flexible manipulator.

Parameters	Description	Value
L	Length of the flexible manipulator	0.6 m
m	Mass of the end payload	6.7 kg
T	Tension of the flexible manipulator	5 N
E	Elastic Modulus of the flexible manipulator	$6.9\times 10^{10}N/m^{2}$
ρ	Density of the flexible manipulator	$2.767\times 10^{3}kg/m^{3}$
B1	Width of the cross section in the flexible manipulator	$6\times 10^{-2}$ m
B2		$4\times 10^{-2}$ m
H1	Height of the cross section in the flexible manipulator	$3\times 10^{-3}$ m
H2		$2\times 10^{-3}$ m

### Numerical Analysis of Flexible Manipulators Control

**FIGURE 4:**
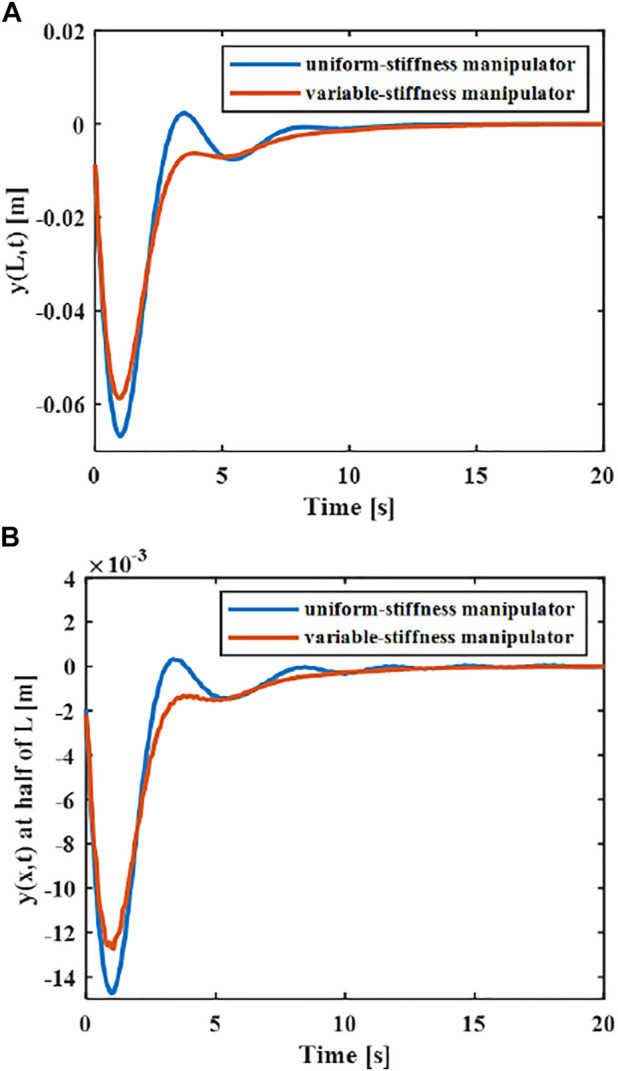
Elastic deformation of different flexible manipulators **(A)** x = L. **(B)** x = L/2.

In order to explore the superiority in the variable-stiffness manipulator, the control effects of two different rigid-flexible coupling manipulator models can be compared. The flexible robotic manipulators connected with a cantilever manner on the central rigid body include a uniform-stiffness robotic manipulator and a variable-stiffness flexible manipulator. The length and quality of the manipulator remains equal. The cross-section height *H* of the uniform-stiffness flexible manipulator is $2\text{.}5\times 10^{-3}$ m and the width *B* is $5\times 10^{-2}$ m. The elastic displacement changes of the middle and end of the uniform and variable-stiffness flexible manipulator are shown in Figure 4.

With the same controller and the same material properties of the manipulator, the maximum amplitude of the end of the variable-stiffness manipulator is 0.058 m, while the maximum amplitude of the end of the uniform-stiffness manipulator is 0.065 m. It can be found that the maximum elastic displacement of the variable stiffness manipulator is smaller.

### Design of Motion Trajectory

According to the dynamic model of the variable-stiffness flexible manipulator system, it can be seen that the elastic vibration of the flexible manipulator is related to the joint angular displacement, angular velocity and angular acceleration. The trajectory planning of the flexible manipulator is the process of moving from the initial state to the target state within a certain period of time. According to the dynamic equation of the flexible manipulator system, the relationship between elastic vibration and motion trajectory is established. Therefore, the vibration suppression control of the flexible manipulator system can be performed by the method of joint trajectory planning, and the residual vibration of the system can be reduced.

In order to avoid excessive elastic vibration during the movement towards the flexible manipulator system, not only the trajectory of the system is required to be continuous, but also the first and second derivatives of the trajectory function are also continuous. When trajectory planning is carried out, the following three conditions must be met on the premise of meeting the specified time for the starting position on the target position:

1) The trajectory is smooth and continuous and maintains a monotonous increase or decrease.
2) The track speed and acceleration are smooth and continuous, and do not exceed the maximum limit value.
3) The following constraints need to be met:

$$\left\{ \begin{array}{l} \theta \left(t_{0}\right)=\theta_{0},\;\theta \prime \left(t_{0}\right)=0,\;\theta \prime \prime \left(t_{0}\right)=0\\ \theta \left(t_{b}\right)=\theta_{b},\;\theta \prime \left(t_{b}\right)=0,\;\theta \prime \prime \left(t_{b}\right)=0 \end{array}\right.$$ (30)

Where $t_{0}$ and $t_{b}$ are the starting time and ending time respectively, $\theta_{0}$ and $\theta_{b}$ are the starting position and ending position of the joint respectively, θ′ and θ′′ are the joint angular velocity and acceleration respectively.

At present, the common motion trajectory curves that meet the above constraints mainly include polynomial of degree five, cycloid and exponential functions (Biagiotti and Melchiorri, 2009). The polynomial of degree five, cycloid and exponential functions were taken as the motion trajectory, and compare the elastic vibration generated by the flexible manipulator under different motion trajectories.

$$\left\{ \begin{array}{l} \theta_{1}\left(t\right)=\left(\theta_{b}-\theta_{0}\right)\left[6{\left(\frac{t}{T_{E}}\right)}^{5}-15{\left(\frac{t}{T_{E}}\right)}^{4}+10{\left(\frac{t}{T_{E}}\right)}^{3}\right]+\theta_{0}\\ \theta_{2}\left(t\right)=\left(\theta_{b}-\theta_{0}\right)\left[\frac{t}{T_{E}}-\frac{1}{2\pi }\sin\left(\frac{2\pi t}{T_{E}}\right)\right]+\theta_{0}\\ \theta_{3}\left(t\right)=\left(\theta_{b}-\theta_{0}\right)\left[\frac{1}{2}+v_{c}\int_{0}^{\tau }e^{-\sigma \cdot \frac{\sin^{2}\pi \tau }{{\left|\cos\pi \tau \right|}^{\lambda }}}d\tau \right]+\theta_{0}\; \end{array}\right.$$ (31)

Where $T_{E}$ is the trajectory movement time, σ and λ are free parameters, and $\tau =\left(\frac{t}{T_{E}}-0.5\right)$ .

**FIGURE 5:**
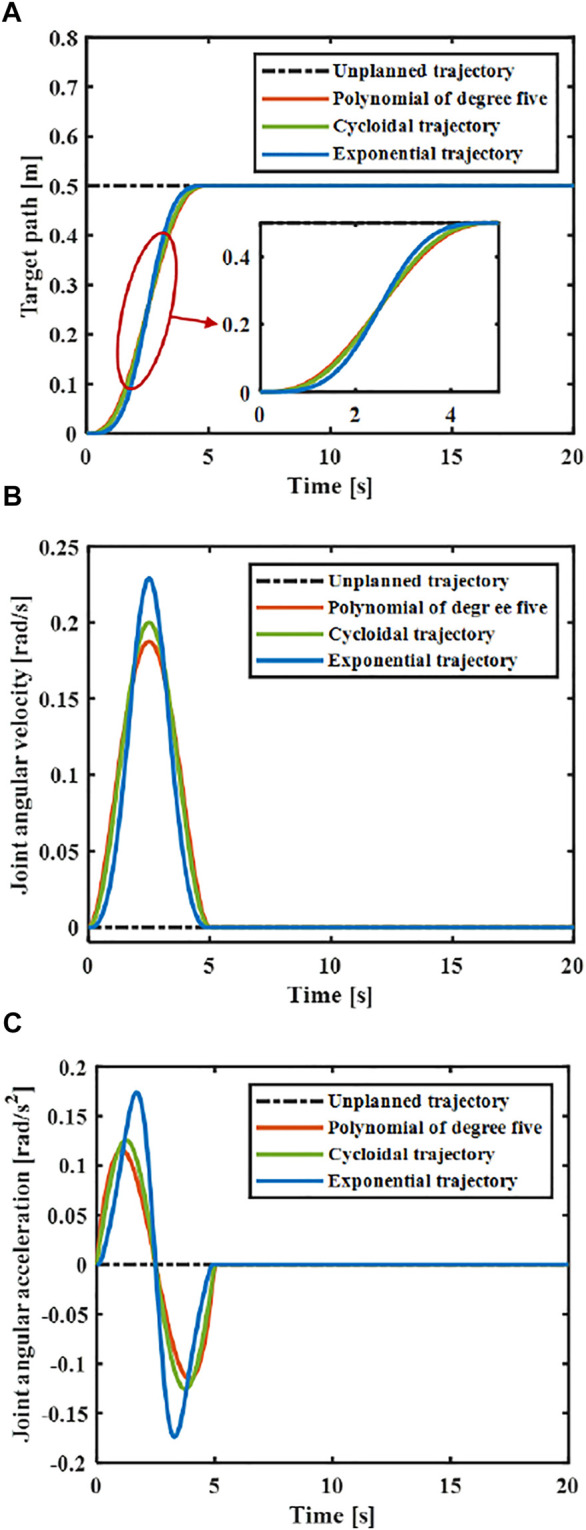
Comparison of different designed trajectories. **(A)** Joint angular displacement. **(B)** Joint angular velocity. **(C)** Joint angular acceleration.

It is assumed that $t_{b}=20s$ , $T_{E}=5s$ , $\theta_{0}=0$ , and $\theta_{b}=0.5$ , the joint angular displacement, angular velocity and angular acceleration of the flexible manipulator under different trajectories can be obtained. As shown in Figure 5, the designed trajectory meets the above constraints.

### Numerical Analysis of Control Based on Different Design Trajectories

**FIGURE 6:**
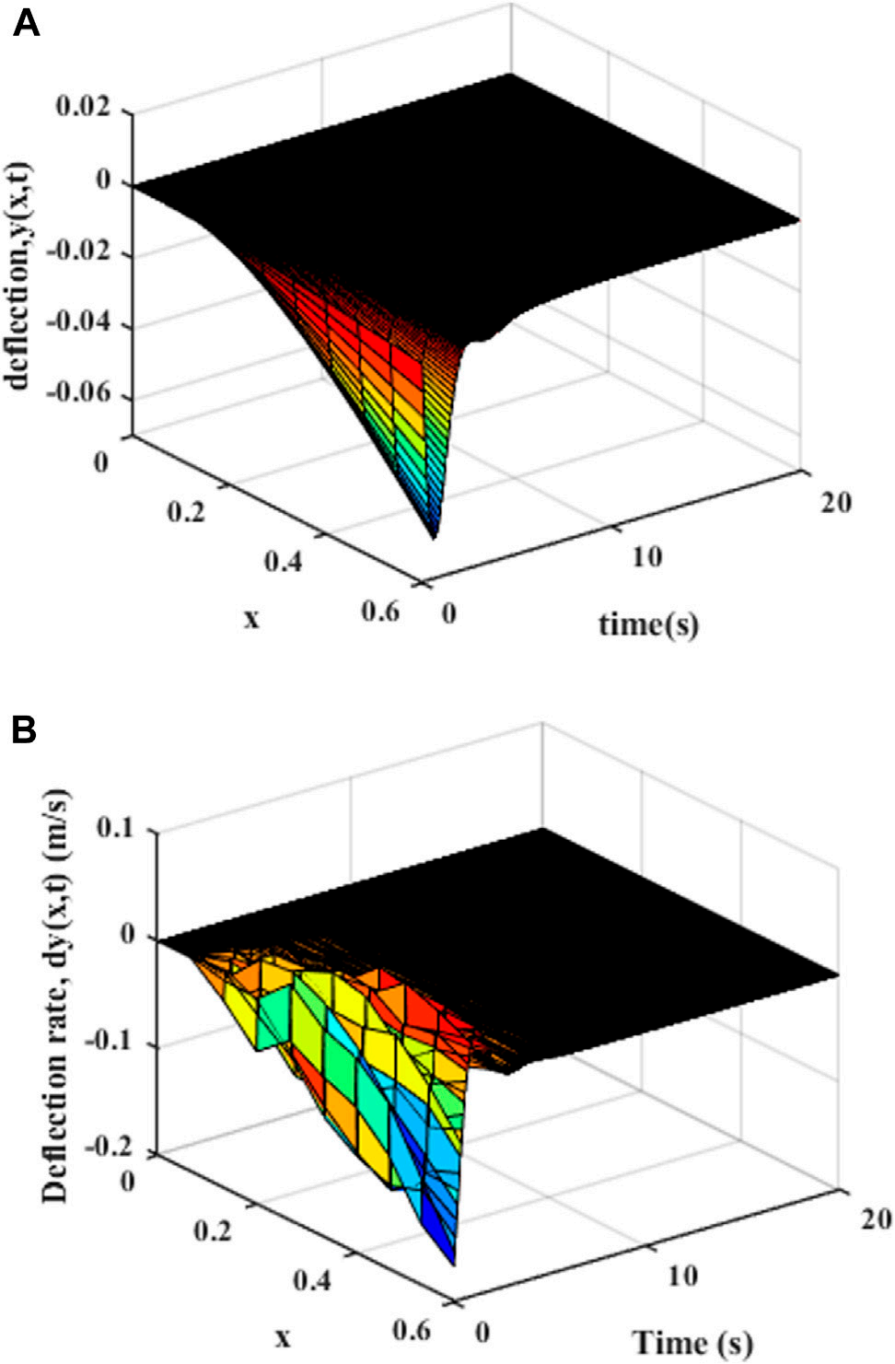
Movement without trajectory planning: **(A)** Elastic deformation. **(B)** Rate of change of elastic deformation.

Since there is no design movement trajectory, as shown in Figure 6, it can be found that the variable-stiffness flexible manipulator system obviously has greater vibration, where the deflection of the variable-stiffness flexible manipulator system reaches to 0.058 m. The system reached a steady state after about 10 s.

**FIGURE 7:**
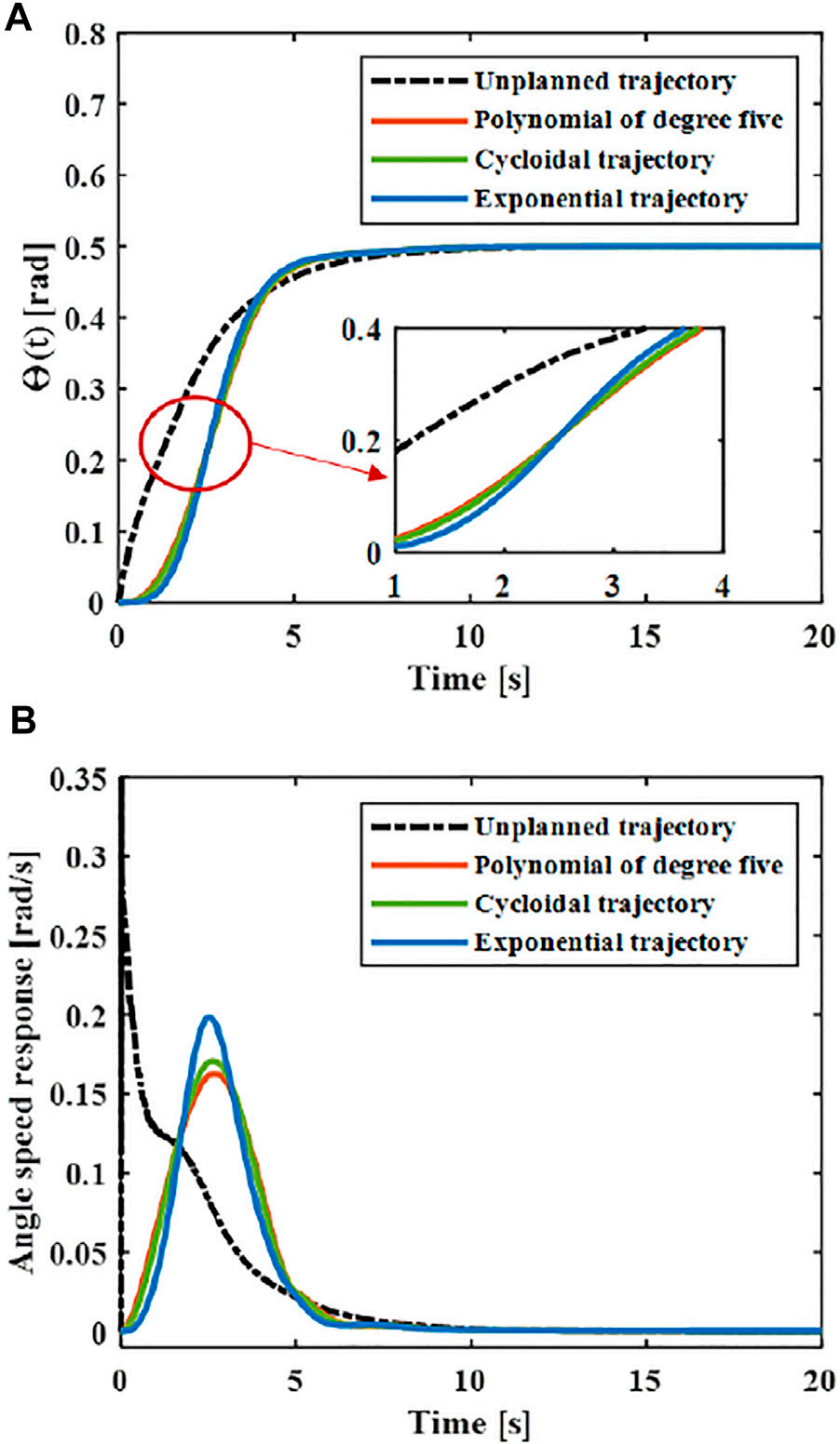
Trajectory tracking: **(A)** Angle. **(B)** Angular speed.

The central rigid body joint performs trajectory tracking based on the designed controller according to the trajectory Eq. 31, and the corresponding joint angle and acceleration changes can be obtained. As shown in Figure 7, the greater the maximum acceleration of the motion trajectory, the greater the fluctuation of the joint angle. The smaller the acceleration when near the target position, the faster the system will stabilize.

**FIGURE 8:**
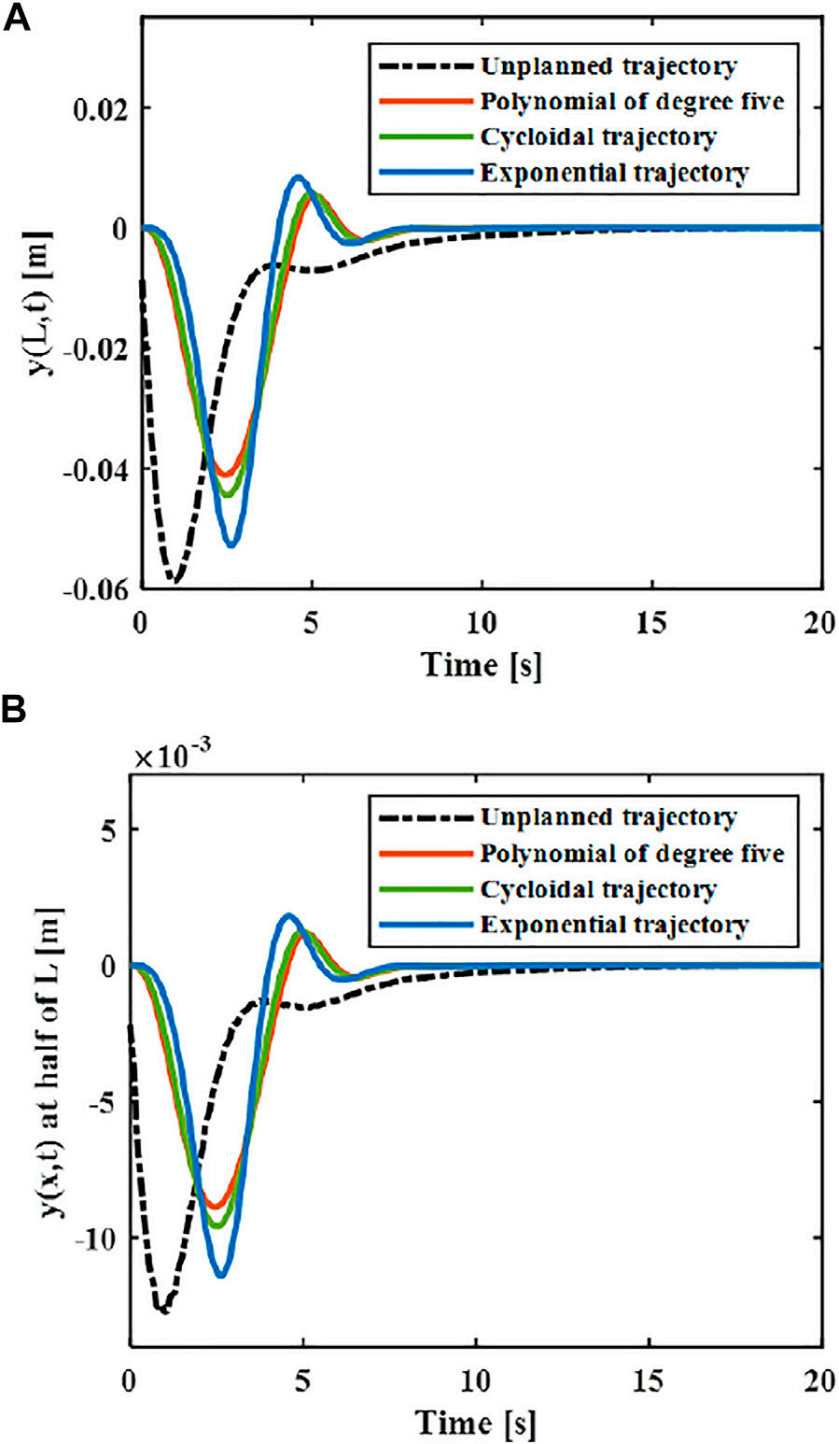
Elastic deformation with the trajectory planning **(A)** x = L. **(B)** x = L/2.

The elastic displacement changes of the middle and end of the variable-stiffness flexible manipulator under the designed trajectory are shown in Figure 8. The flexible manipulator has large elastic vibrations in the process of following the trajectory, and there is still a certain degree of residual vibration after the movement. Under different motion trajectories, the elastic vibration changes of the flexible mechanical manipulator are different. The greater the maximum acceleration of the motion trajectory, the greater the maximum elastic displacement generated. The smaller the acceleration when near the target position, the smaller the residual vibration displacement. When there is no trajectory planning, a large elastic displacement will be produced during the movement, and vibration will be produced during the movement, especially after the movement, the vibration takes a long time to recover to a stable state. This situation not only reduces the stability of the system, but also shortens the service life of the flexible manipulator. According to the comparison results, the maximum elastic displacement of the end under the fifth-order polynomial motion trajectory, cycloid motion trajectory and exponential motion is 0.040, 0.044 and 0.056 m. It can be found that the maximum elastic displacement of the flexible manipulator is the smallest when the movement is planned according to the polynomial of degree five trajectory.

**FIGURE 9:**
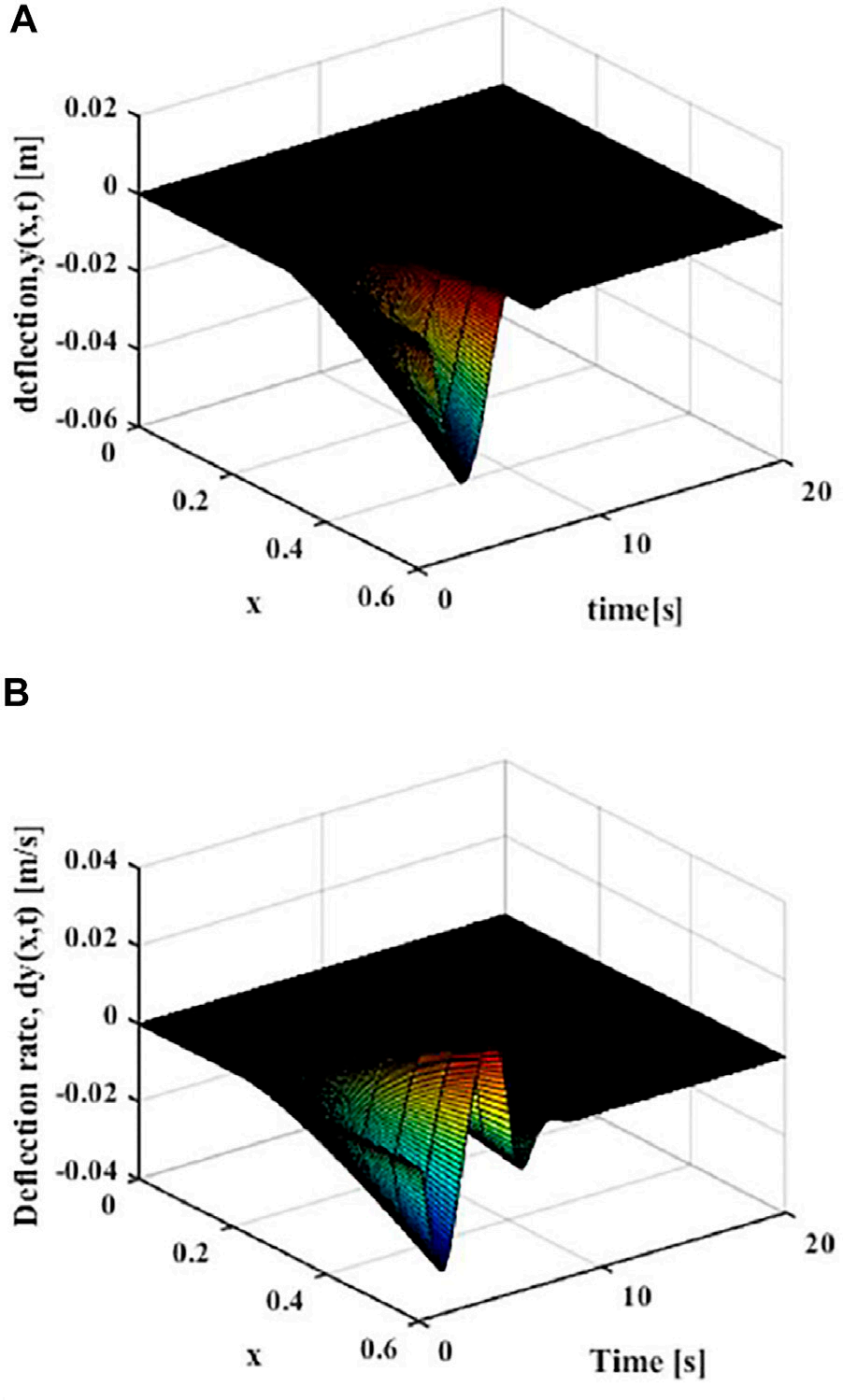
Movement with the polynomial of degree five trajectory (x = L) **(A)** Elastic deflection. **(B)** Elastic deflection rate.

As shown in Figure 9, the distributed elastic deformation and change rate of the manipulator after the polynomial of degree five trajectory movement can be obtained. It can be found that the vibration of the variable-stiffness flexible manipulator system is obviously reduced, where the maximum deflection rate of the variable-stiffness flexible manipulator system reaches to 0.028 m. The system reached a steady state after about 5 s.

## Optimization of Vibration Suppression Trajectory

According to the vibration suppression results after trajectory planning, it can be found that vibration at the end of the variable-stiffness flexible manipulator after joint angular motion is still large. In order to make the mechanical arm system have a small residual vibration during movement and reduce it to zero in a short time, and make the end of the system reach the target position quickly and accurately, the above ideal trajectory needs to be optimized.

### Optimization Target of Vibration Reduction

In view of the characteristics of the flexible manipulator system, considering the conservation of energy, the non-conservative force is used to express the total energy consumed by the system during the movement. In order to measure the amount of elastic vibration of the flexible manipulator during the movement and after the movement, a suppression indexed including the elastic displacement of the end during the movement and the residual vibration displacement of the end after the movement is proposed. Therefore, the objective function can be obtained as

$$J=r_{1}\int_{0}^{4T_{E}}\left|u\left(x,t\right)\theta \prime \left(t\right)+F\left(x,t\right)z\prime \left(L,t\right)\right|dt+r_{2}\int_{0}^{4T_{E}}y^{T}\left(l_{L},t\right)y\left(l_{L},t\right)dt+r_{3}\int_{0}^{4T_{E}}\left|dis\left(t\right)\right|dt$$ (32)

Where $y\left(l_{L},t\right)$ is the elastic displacement of the end of the flexible manipulator, $r_{1}$ , $r_{2}$ and $r_{3}$ are the weighting factors of the three terms in the formula, and $r_{1}+r_{2}+r_{3}=1$ . *u(x,t)* is the control input signal, and dis(*t*) is the distance between the actual trajectory and the ideal trajectory. When trajectory planning is performed to ensure that $y\left(l_{L},t\right)$ is minimum, the purpose of vibration suppression of the flexible manipulator system can be achieved through trajectory planning.

### Trajectory Optimization Based on CADE Algorithm

**FIGURE 10:**
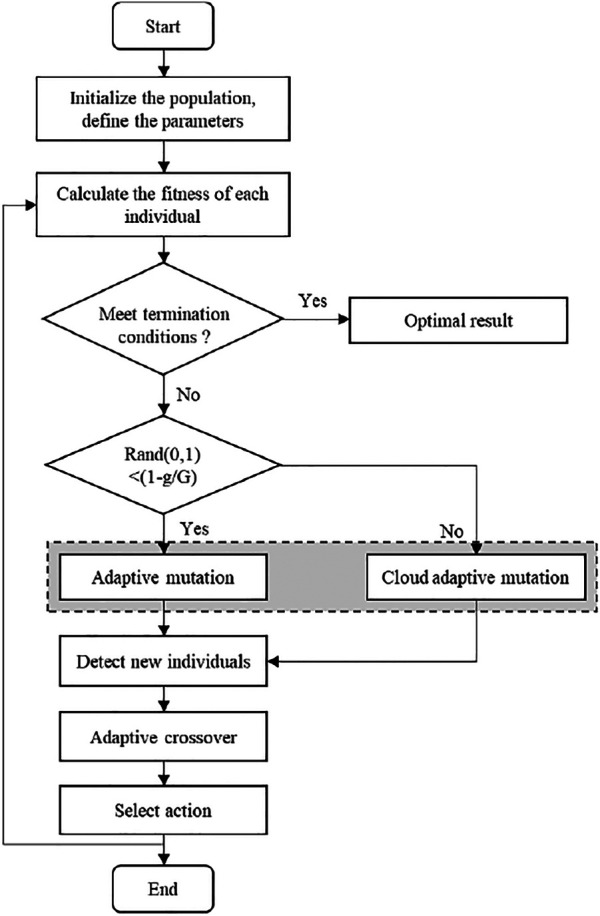
CADE algorithm calculation flow chart.

The differential evolution (DE) optimization algorithm is a bionic intelligent algorithm that simulates the biological evolution mechanism of nature. The realization mechanism is to randomly reorganize the temporary individuals generated by the individual differences in the population to complete the population evolution. However, the mutated individuals are selected randomly, which increases the randomness of the algorithm, which leads to randomness in the optimization direction, and reduces the convergence speed. The cloud adaptive differential evolution (CADE) optimization algorithm uses cloud mutation operation, and new individuals are generated near the best individuals produced by the previous generation, which not only improves the convergence speed, but also maintains the characteristics of the best individuals. To ensure the randomness of basic mutation operations and the stable tendency of cloud mutation operations, the two methods are combined to perform mutation operations. The optimization algorithm has stronger robustness and convergence, and has a good effect on solving numerical optimization problems. The CADE algorithm flow chart is shown in Figure 10.

In order to obtain the optimal trajectory, the ideal trajectory (polynomial of degree five) is optimized to minimize the objective function Eq. 32 based on the CADE optimization algorithm. The algorithm introduces a cloud model that can generate cloud droplets with a stable tendency. During the evolution process, it can target the optimal individual, perform adaptive positioning of the global optimal solution, and improve the convergence speed. The mutation operation of the CADE algorithm is completed by the normal cloud generator, and the mutation factor and crossover factor are adaptively adjusted during the evolution process to ensure the diversity of new individuals in the early stage and the convergence in the later stage.

After the ideal trajectory is optimized by the CADE algorithm, a set of best deviations can be obtained, and then the best discrete trajectory is obtained as

$${\bar{\theta }}_{op}=\left[{\bar{\theta }}_{op,0},{\bar{\theta }}_{op,0},...,{\bar{\theta }}_{op,2n-1},{\bar{\theta }}_{op,2n}\right]$$ (33)

In order to obtain the continuous optimal trajectory, the cubic spline interpolation method is used to interpolate the discrete trajectory. The interpolation condition can be defined as

$$\left\{ \begin{array}{l} \theta_{op}\left(0\right)={\bar{\theta }}_{op,0}=\theta_{0}\\ \theta_{op}\left(T_{E}\right)={\bar{\theta }}_{op,2n}=\theta_{d}\\ \theta \prime_{op}\left(0\right)=\theta \prime_{0}=0\\ \theta \prime_{op}\left(T_{E}\right)=\theta {'}_{d}=0 \end{array}\right.$$ (34)

Where the interpolation node is

$$\theta_{op}\left(t_{j}\right)={\bar{\theta }}_{op,j},\;t_{j}=\frac{j}{2n}T_{E},\;j=1,2,...,2n-1$$ (35)

**FIGURE 11:**
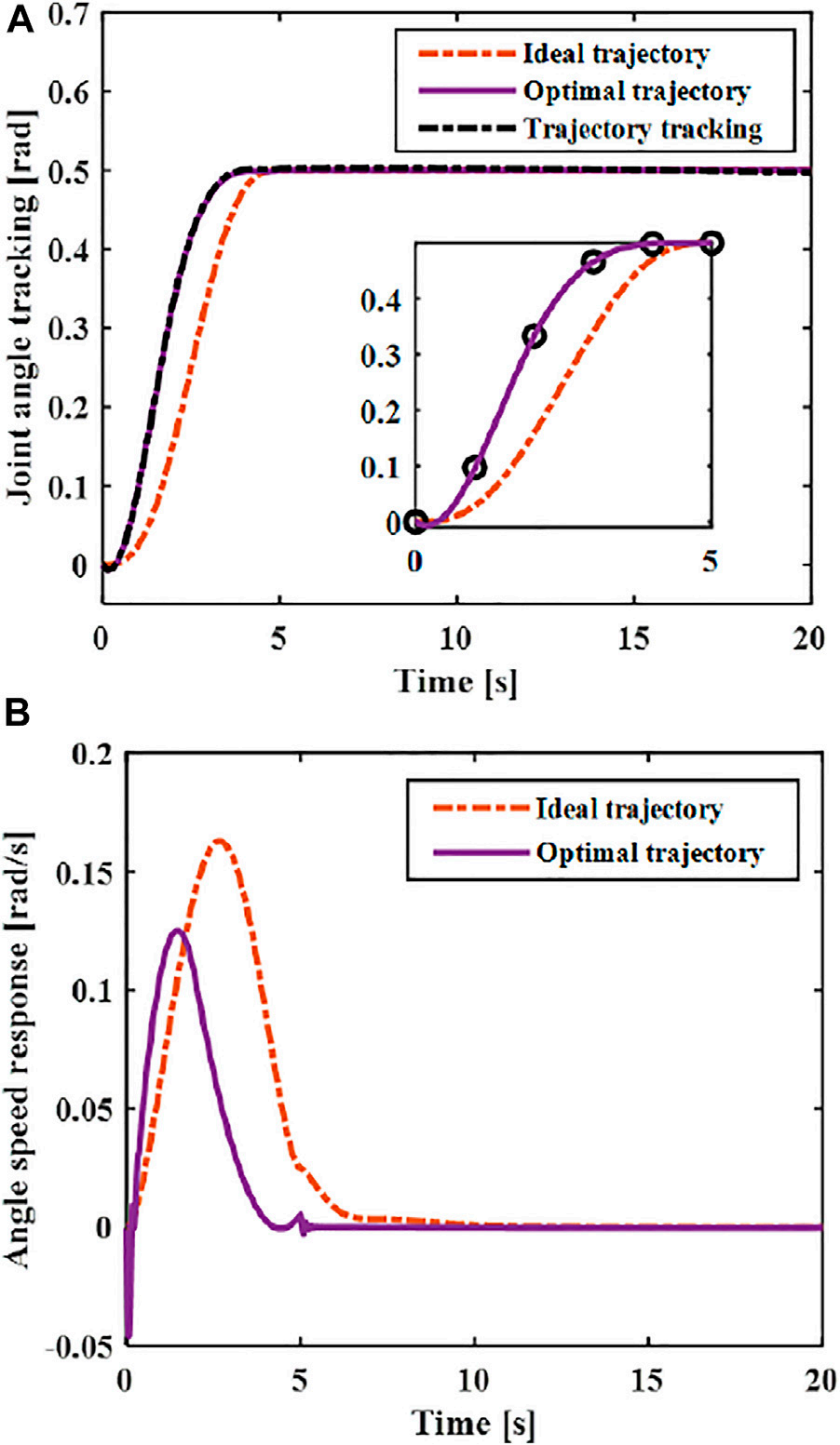
Comparison of ideal trajectory and optimized trajectory **(A)** Angle **(B)** Angle speed.

The continuous function obtained by interpolation is used as the optimal trajectory of the joint. The designed controller Eq. 16 is used to track the optimal trajectory. As shown in Figure 11, the optimal vibration suppression trajectory curve and its speed curve can be obtained through optimization. Through comparing with the ideal trajectory, it can be seen that the optimized trajectory and its speed after optimization meet the boundary constraints, so the optimized trajectory meets the vibration suppression requirements. By comparing with the ideal motion trajectory, it can be seen that the maximum speed of the optimized vibration suppression trajectory are less than that of the ideal trajectory (polynomial of degree five).

**FIGURE 12:**
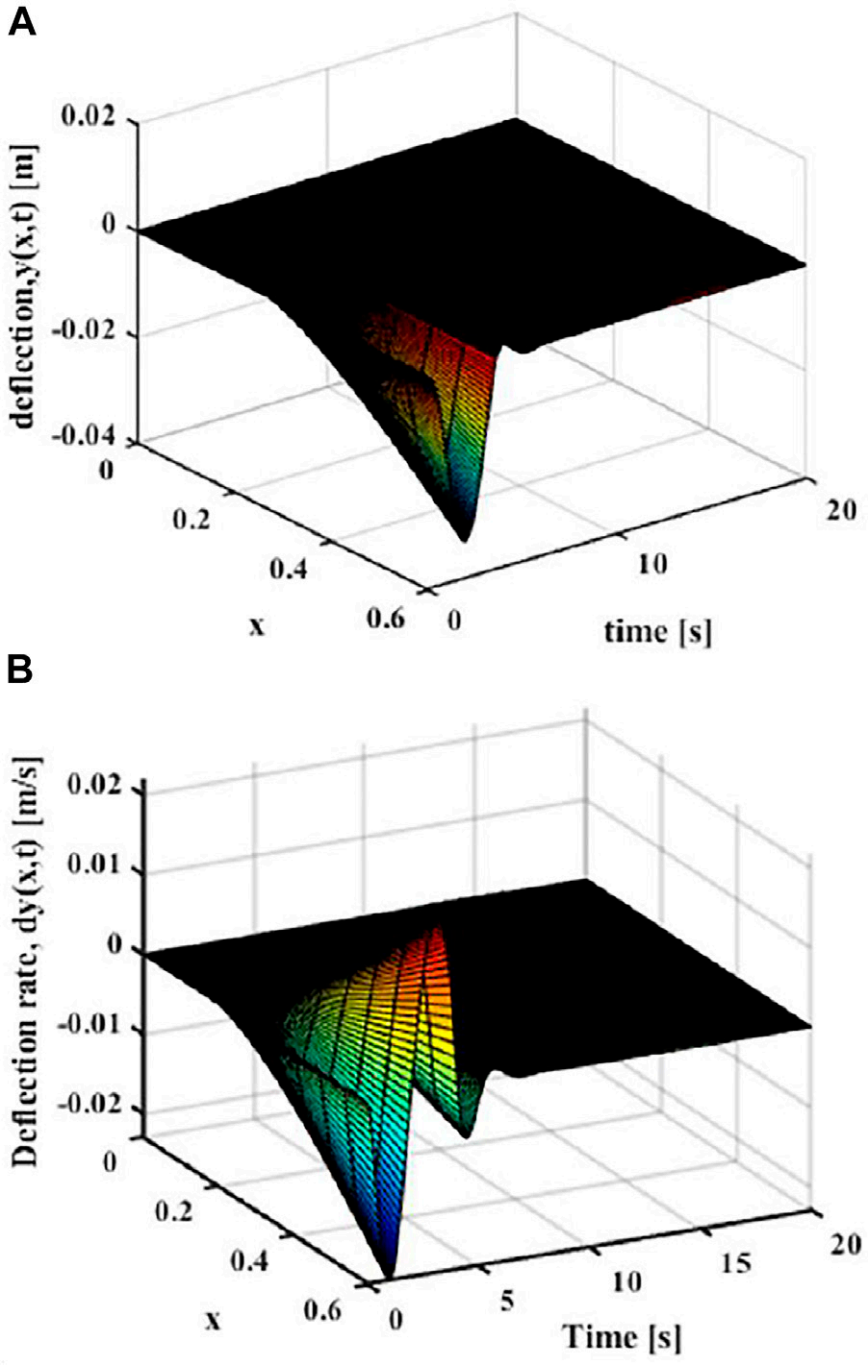
Movement with the optimized trajectory (*x* = *L*) **(A)** Elastic deflection. **(B)** Elastic deflection rate.

Then the effect of trajectory vibration suppression after unverified optimization can be obtained. As shown in Figure 12, it can be seen that the maximum elastic displacement of the end of the flexible manipulator under the optimized vibration suppression trajectory is 0.033 m, the maximum elastic deflection stabilizes after 4 s.

**FIGURE 13:**
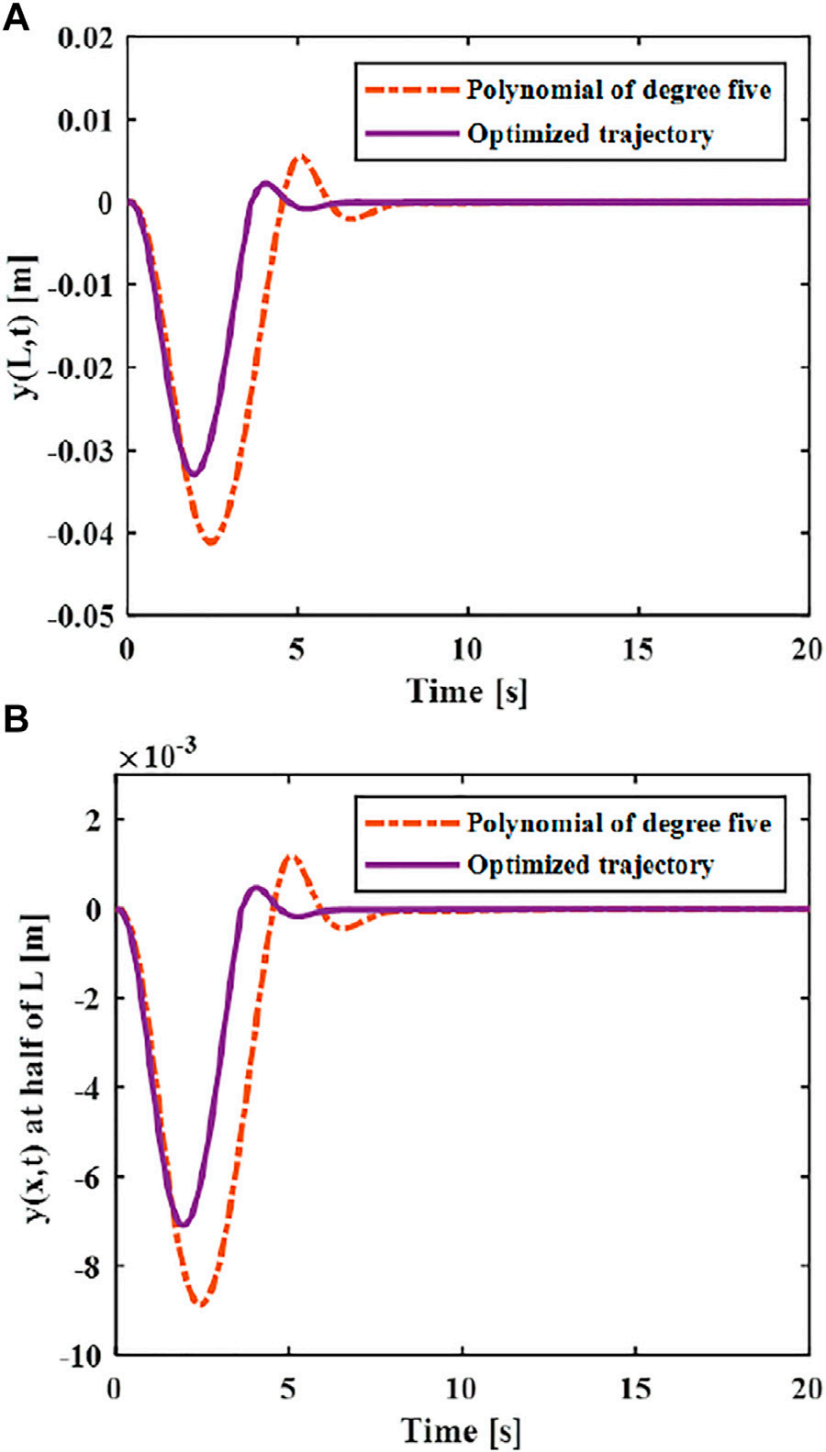
Elastic deflection of ideal trajectory and optimized trajectory **(A)** *x* = *L*. **(B)** *x* = *L*/2.

The vibration suppression effect after the optimized trajectory and the ideal trajectory are compared. As shown in Figure 13, it can be seen that he flexible manipulator moves under the optimized trajectory, and the elastic displacement of the end is smaller than the ideal trajectory during the movement, and the maximum elastic displacement ratio between the optimal trajectory and the ideal trajectory is 0.8:1. Compared with the elastic vibration during the movement of the flexible manipulator, the vibration at the end of the flexible manipulator is suppressed to a greater extent of the optimal vibration suppression trajectory. After the movement is completed, the flexible manipulator can quickly return to stable state, which is 1 s faster than the ideal trajectory.

## Conclusion

In this work, the vibration control problem of a variable-stiffness flexible manipulator with boundary input is studied. Taking into account the coupling characteristics between the central rigid body and the robot manipulator, using Hamilton’s principle, the PDE dynamic model of the flexible system is derived. It is worth mentioning that all analyses are based on the original PDE model. Simulation studies shows that the elastic deflection of the variable-stiffness flexible manipulator after trajectory planning is significantly reduced, and the system stabilizes in a short period of time. Then the ideal trajectory is optimized based on the CADE algorithm. The variable stiffness manipulator system under the optimized trajectory tends to stabilize and converge after 5 s. The proposed trajectory planning method not only improves the stability and positioning accuracy of the variable stiffness robot manipulator system, but also has a better vibration reduction effect. This study did not consider the influence of materials on the elastic deformation of the manipulator. In the future, the controller design for the variable-stiffness robotic flexible manipulator in the presence of different disturbances will be studied.

## Data Availability

The original contributions presented in the study are included in the article/supplementary material, further inquiries can be directed to the corresponding author.
